# Reconstructing COVID-19 incidences from positive RT-PCR tests by deconvolution

**DOI:** 10.1186/s12879-023-08667-1

**Published:** 2023-10-11

**Authors:** Mengtian Li, Jiachen Li, Ke Wang, Lei M. Li

**Affiliations:** 1 National Center of Mathematics and Interdisciplinary Sciences, Academy of Mathematics and Systems Science, Chinese Academy of Sciences, Beijing, 100190 China; 2 Capital University of Economics and Business, Beijing, 100070 China; 3 University of Chinese Academy of Sciences, Beijing, 100049 China

**Keywords:** COVID-19, Daily incidences, RT-PCR test, Delay function, Richard-Lucy deconvolution

## Abstract

**Background:**

The emergency of new COVID-19 variants over the past three years posed a serious challenge to the public health. Cities in China implemented mass daily RT-PCR tests by pooling strategies. However, a random delay exists between an infection and its first positive RT-PCR test. It is valuable for disease control to know the delay pattern and daily infection incidences reconstructed from RT-PCR test observations.

**Methods:**

We formulated the convolution model between daily incidences and positive RT-PCR test counts as a linear inverse problem with positivity restrictions. Consequently, the Richard-Lucy deconvolution algorithm was used to reconstruct COVID-19 incidences from daily PCR tests. A real-time deconvolution was further developed based on the same mathematical principle. The method was applied to an Omicron epidemic data set of a bar outbreak in Beijing and another in Wuxi in June 2022. We estimated the delay function by maximizing likelihood via an E-M algorithm.

**Results:**

The delay function of the bar-outbreak in 2022 differs from that reported in 2020. Its mode was shortened to 4 days by one day. A 95% confidence interval of the mean delay is [4.43,5.55] as evaluated by bootstrap. In addition, the deconvolved infection incidences successfully detected two associated infection events after the bar was closed. The application of the real-time deconvolution to the Wuxi data identified all explosive incidence increases. The results revealed the progression of the two COVID-19 outbreaks and provided new insights for prevention and control strategies, especially for the role of mass daily RT-PCR testing.

**Conclusions:**

The proposed deconvolution method is generally applicable to other infectious diseases if the delay model can be assumed to be approximately valid. To ensure a fair reconstruction of daily infection incidences, the delay function should be estimated in a similar context in terms of virus variant and test protocol. Both the delay estimate from the E-M algorithm and the incidences resulted from deconvolution are valuable for epidemic prevention and control. The real-time feedback is particularly useful during the epidemic’s acute phase because it can help the local disease control authorities modify the control measures more promptly and precisely.

**Supplementary Information:**

The online version contains supplementary material available at 10.1186/s12879-023-08667-1.

## Introduction

The emergence of new COVID-19 variants over the past three years has posed a serious challenge to public health. From August 2021 to the fall of 2022, the Chinese public health department adopted a dynamic zero-COVID policy that aimed to eliminate local transmission through rigorous testing, tracing, and isolation. Once a local outbreak of the epidemic occurs, it is important for decision-makers to get an accurate and rapid analysis of the epidemic, including the number of people who have been infected, the scope of infection, and the speed of transmission. In order to get real-time infection information, cities or regions in China implemented mass daily RT-PCR (Reverse Transcription Polymerase Chain Reaction) tests by the pooling strategy. However, the RT-PCR test, though recognized as the gold standard in COVID-19 diagnosis [1], has a high false-negative rate during the first few days after infection [2]. The delay between one’s infection and his First Positive RT-PCR Test (FPRT) is a random variable, whose distribution depends on factors such as the pathological characteristics of the new coronavirus variant, the testing strategy, and the reporting pipeline arrangement. Three questions arise naturally: 1. Can we estimate the delay pattern with accuracy evaluation? 2. How can we reconstruct the daily infection incidences from the daily positive PCR test series? 3. Can we find some insights from the above two results to guide the policy in the case of an epidemic outbreak?

In the literature, Goldstein et al. [3] utilized Richard-Lucy deconvolution [4, 5] to reconstruct the incidence curves for the 1918 influenza epidemic from a recorded daily death curve and a time-to-death distribution. In the scenario of COVID-19, Maria Jahja [6] considered the delay between symptom onset and reporting dates by the local public health authorities.

In contrast, we focused on the delay from infection-by-contact to one’s FPRT date in this article. Specifically, we assume the availability of mass daily RT-PCR tests. During 2022 in China, mass daily RT-PCR tests in relevant regions were a common practice once an outbreak was reported in a city. This study used the daily FPRT counts from two Omicron outbreaks, one in Beijing, and the other in Wuxi.

We represent the observed FPRT series as a convolution of the infection series and the delay distribution in a COVID-19 epidemic. The reconstruction of the daily infection incidences is nothing but deconvolution, which is generally ill-posed as an inverse problem. Since both counts and delay distribution are nonnegative, we formulated the problem as a linear inverse problem with positivity restrictions (LININPOS). The non-negativity mitigates the ill-posedness greatly by converting the problem into a convex optimization one [7, 8]. Moreover, by the same mathematical principles, we developed a real-time deconvolution algorithm that enables us to reconstruct daily incidence counts timely as an outbreak progresses. We used the proposed method to analyse public data from a bar-related Omicron outbreak in Beijing in June 2022. First, we estimated, by maximizing likelihood, the delay function based on 258 bar-visitors. Then the estimated delay function was put into the deconvolution algorithm to reconstruct the daily incidences of the other bar-involver group. The inferences made from the outbreak of mere 393 cases unveiled insights into the transmission and PCR test characteristics of the Omicron virus. Furthermore, we applied the real-time deconvolution algorithm to the epidemic in Wuxi City, which occurred immediately after the bar-outbreak in Beijing.

The deconvolution approach potentially has several real-world applications. First, the estimate of the delay function is a key epidemiological parameter relating to the incubation of the virus variant. Second, the daily incidences reconstructed from deconvolution would help the local disease control authorities identify significant infection events retrospectively and understand the transmission pattern. Third, the real-time deconvolution is particularly useful during the epidemic’s acute phase because it can help the local disease control authorities modify the control measures swiftly. Finally, the code of the approach is publicly-available as Supplementary materials.

## Methods

### The convolution model

Consider an epidemic chain in which a total of *N* patients are reported within *n* days. Notice that only patients who have at least one positive RT-PCR test are considered. Denote the series of daily FPRT count as $\textbf{G}=(G_1,G_2,\dots ,G_n)$, and the series of daily infection count as $\textbf{F}=(F_1,F_2,\dots ,F_n)$. The former is observed while the latter is not. For each patient indexed by *k*, denote his infection date and FPRT date respectively by random variables $\textbf{X}(k)$ and $\textbf{Y}(k)$. Suppose that for all infected individuals, their delay days from infection to testing positive, follow the same probability distribution, namely,

1 $$\begin{aligned} P(\textbf{Y}(k)=s+t|\textbf{X}(k) = s)\triangleq w_t,\quad\forall s \in \{1,2,\dots ,n\},\quad t=0,1,\dots , m, \end{aligned}$$

where *m* is the maximum delay. By the calculation of total probability, we have

2 $$\begin{aligned} P(\textbf{Y}(k) = j)=\sum \limits _{t=0}^m P(\textbf{X}(k)=j-t)P(\textbf{Y}(k)=j|\textbf{X}(k) = j-t),\quad j=1,2,\dots ,n. \end{aligned}$$

If we write the probability as the expectation of an indicator variable, the above Eq. (2) becomes: $E[I_{(\textbf{Y}(k) = j)}]=\sum _{t=0}^m E[I_{(\textbf{X}(k)=j-t)}]w_t$. Summing over all individuals $k\in \{1,2,\dots ,N\}$, we get $E[G_j]=\sum _{t=0}^m E[F_{j-t}]w_t$. Let $\textbf{w}\triangleq (w_{t})$, the simple convolution equation is established:

3 $$\begin{aligned} E[\textbf{G}]=E[\textbf{F}]* \textbf{w} , \end{aligned}$$

where $*$ denotes the convolution operator.

### Estimate the daily incidences by the Richard-Lucy deconvolution

A fair estimate of $\textbf{F}$ is its expectation. The expectation of $\textbf{G}$ in (3) can be replaced by its observation. When the delay distribution $\textbf{w}$ is known, we can reconstruct $\textbf{F}$ by deconvolution. Since all elements of $F_i, G_j, w_t$ are non-negative, this is a typical LININPOS problem defined by Vardi and Lee [7]. Remember $\sum _i F_i = \sum _j G_j = N$ and $\sum _t w_{t}=1$, normalizing both sides of (3) by dividing *N* makes them probability mass functions over day indices $\{1,2,\dots ,n\}$. Denote

4 $$\begin{aligned} f_i=\frac{F_i}{N},\quad g_j=\frac{G_j}{N}, \quad i,j=1,..,n. \end{aligned}$$

Denote the reconstructed values by $\hat{\textbf{F}}$. Due to variations, the difference between $\textbf{G}$ and $\hat{\textbf{F}}* \textbf{w}$ are not necessarily zero. A natural measure of goodness of fit is the Kullback-Leibler divergence proposed in [7], namely, we solve $\hat{\textbf{F}}$ by minimizing the following:

5 $$\begin{aligned} D_{KL}(\textbf{G}||\hat{\textbf{F}}* \textbf{w}) \triangleq \sum \limits _{j=1}^n G_j\log \left( \frac{G_j}{\sum _{i} \hat{F}_iw_{j-i}}\right) . \end{aligned}$$

Using a heuristic approach to optimizing the divergence, Vardi and Lee [7] proposed an E-M algorithm, which turned out to be nothing but the Richard-Lucy deconvolution. Li and Speed [8] proved its convergence directly by showing that each iteration reduces the Kullback-Leibler divergence, which is a convex function defined over a convex set. Since it is an optimization problem subject to nonnegativity constraints, they provided the Kuhn-Tucker condition that the minimizer of (5) satisfies (see Supplementary Note S3). The Richad-Lucy algorithm that we estimate $\hat{\textbf{F}}$ is as follows.

**Figure:**
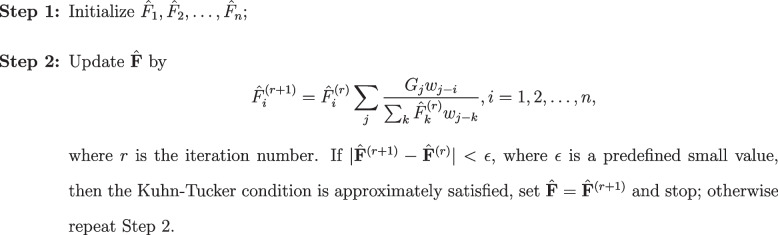
**Algorithm 1** The Richard-Lucy deconvolution algorithm

### Real-time deconvolution

Crucial to infectious disease control is the availability of real-time incidence counts of the current infections, particularly during the pandemic’s acute phase. However, Algorithm 1 needs the complete FPRT series $\{G_j , j = 1, 2, \cdots , n\}$ so that the detected infection events can only be traced and investigated towards the end of an outbreak. To have a more timely tool, we further proposed a real-time deconvolution algorithm by a modification to the Richard-Lucy algorithm.

In this subsection, we consider a more practical assumption in which only the FPRT counts up to the *K*-th $(K<n)$ day are known. We denote the truncated FPRT series as $\textbf{G}_{1:K} = (G_1,\dots ,G_K)$. In this case, the equation between the infection and the FPRT becomes:

$$\begin{aligned} \sum \limits _{j=1}^K G_j = \sum \limits _{j=1}^K\sum \limits _{i=1}^{j} F_iw_{j-i} , \end{aligned}$$

Let $N_K\triangleq \sum _{j=1}^K G_j$, which denotes the sum of positive cases reported up to day *K*. Let

$$\begin{aligned} f_i = \frac{F_i}{N_K}, \quad g_j = \frac{G_j}{N_K},\quad i,j=1,\dots ,K. \end{aligned}$$

Then the normalized parameters $\textbf{g}_{1:K}=(g_1,\dots ,g_K),\textbf{f}_{1:K}=(f_1,\dots ,f_K),\textbf{W}=(w_t)$ form the following equation:

6 $$\begin{aligned} \sum \limits _{i=1}^K \left( \sum \limits _{t=0}^{K-i} w_t\right) f_i=\sum \limits _{j=1}^K g_j = 1. \end{aligned}$$

Denote the first *K* terms of $\hat{\textbf{F}}$ by $\hat{\textbf{F}}_{1:K}$. Accordingly, we solve $\hat{\textbf{F}}_{1:K}$ by minimizing the following function subject to the condition (6):

$$\begin{aligned} D_{KL}\left(\textbf{G}_{1:K}||\hat{\textbf{F}}_{1:K}* \textbf{w}\right) \triangleq \sum \limits _{j=1}^K G_j\log \left( \frac{G_j}{\sum _{i} \hat{F}_iw_{j-i}}\right) . \end{aligned}$$

Similarly, we derived the Kuhn-Tucker condition (details *c.f.* Supplementary Note S4), which led to the deconvolution algorithm that estimates $\hat{\textbf{F}}_{1:K}$ as follows:

**Figure:**
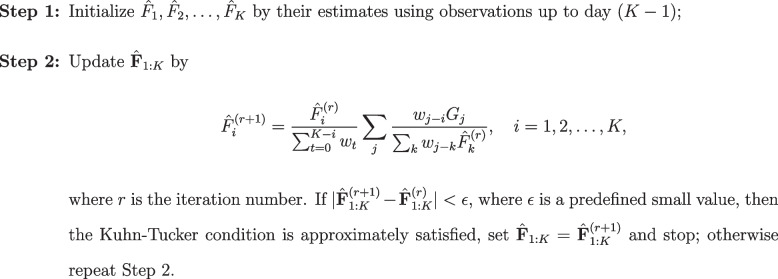
**Algorithm 2** The real-time deconvolution algorithm on day *K*

Compared to Algorithm 1, in the denominator of the iterative formula, Algorithm 2 has an extra term $\left( \sum _{t=0}^{K-i} w_t\right)$, which depends on the current index *K*. Another modification is that in real-time algorithm, we initialize $\hat{\textbf{F}}_{1:K}$ by the counts deconvolved from observations up to day $(K-1)$.

According to the Omicron delay function estimated from the bar-visitors, the sensitivity of the PCR test is zero up to day 2 after infection, that is, $w_0=w_1=w_2=0$. In other words, on day *K* we have no knowledge about the infection incidences on day $(K-1)$ and $(K-2)$ at all. Consequently, the real-time deconvolution on day *K* can only deconvolve the infection counts up to day $(K-3)$.

## Results

### An Omicron outbreak at Heaven Supermarket Bar in Beijing

An Omicron outbreak occurred at Heaven Supermarket Bar, Beijing, in early June 2022. This event separated itself from other infection events reported earlier and offered a valuable dataset for studying its transmission pattern. Due to the persistent reports of COVID-19 cases since April and the increasing trend in May, the Beijing government imposed certain restrictions on business involving people gathering in relevant regions of the city until PCR-positive counts reached dynamic zero for a week. The restriction was lifted on June 6th; however, on exactly the same day an Omicron outbreak occurred at Heaven Supermarket Bar. The outbreak was single-origin yet explosive in nature and widespread in scope. Since the city was on high alert, measures were taken immediately and the spreading was put down completely within three weeks.

**Fig. 1:**
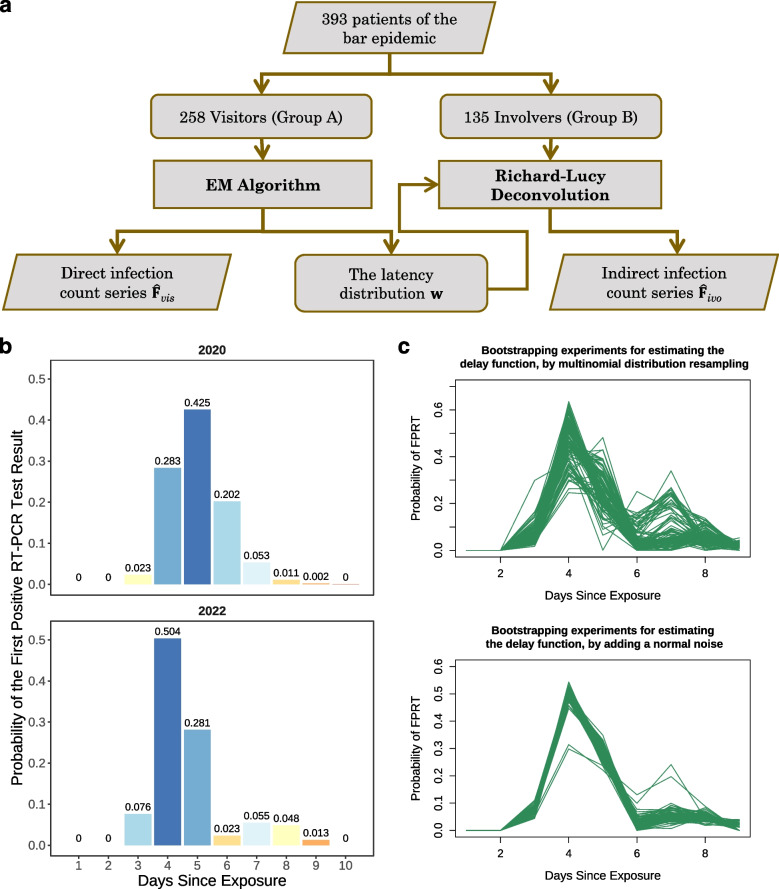
**a** The flow diagram of our research on the bar epidemic. **b** A comparison of the delay distributions between 2020 and 2022. **c** Results for re-estimating the delay distribution with perturbed $\{G_j\}$s. The top plot is perturbed by resampling from the multinomial distribution with the empirical frequencies, and the bottom plot is perturbed by adding a normal noise

We collected the public daily FPRT counts from June 9 to 25 at the official website of Beijing Municipal Health Commission [9] (see Supplementary Table S1). A total of 393 cases had been traced back to the bar. They can further be partitioned into two groups: (A) 258 individuals who were reported to have visited the bar in person, referred to as “visitors”; (B) 135 individuals who had not visited the bar and were infected by previous infectors, referred to as “involvers”. The 258 visitors in group A were known to be infected at the bar from June 6 to 9 whereas they were reported PCR test-positive from June 9 to 15. Based on the data of visitors, we first obtained the maximum likelihood estimate (MLE) of the delay distribution $\textbf{w}$ by an E-M algorithm, which imputed the daily incidences of these visitors $\textbf{F}_{\textit{vis}}$ as well. Next, we reconstructed the daily incidences of the involvers $\textbf{F}_{\textit{inv}}$ in group B from their daily FPRT count series by the Richard-Lucy algorithm. Figure 1a illustrates the flow diagram of the research design.

### The PCR delay distribution of the Omicron variant

In 2020, researchers at Johns Hopkins University [2] combined several published public data sets to study the false-negative rate of RT-PCR test by days since infection. Based on their estimate of the false-negative rate, we computed the corresponding PCR delay distribution of COVID-19 in 2020 as shown at the top in Fig. 1b. In contrast, we estimated the PCR delay distribution of the Omicron variant in Beijing in 2022 based on the Heaven Supermarket Bar epidemic data via an E-M algorithm [10], *c.f.* details in Supplementary Notes.

Comparing the two delay distributions, we can see that the delay between infection and one’s FPRT has been significantly reduced in 2022. Specifically, both the mode and median of delay days have been shortened from 5 to 4 days, and the expectation has decreased from 5.022 to 4.67. The total probability of being PCR-positive within 5 days since exposure increases from $73.2\%$ to $86.1\%$.

A related parameter in epidemiology is the incubation period, which is defined as the interval between the date of transmission and the date of onset of clinical symptoms/signs in the study subjects. A shorter incubation period for the BA.1 Omicron variant in 2022 than that for the Alpha variant in 2021 was demonstrated by previous research [11]. The former was $3.03 \pm 1.35$ days (mean ± SDM) while the latter was $4.94 \pm 2.19$ days. The shorter incubation period and the PCR delay days were consistent for the Omicron variant.

**Fig. 2:**
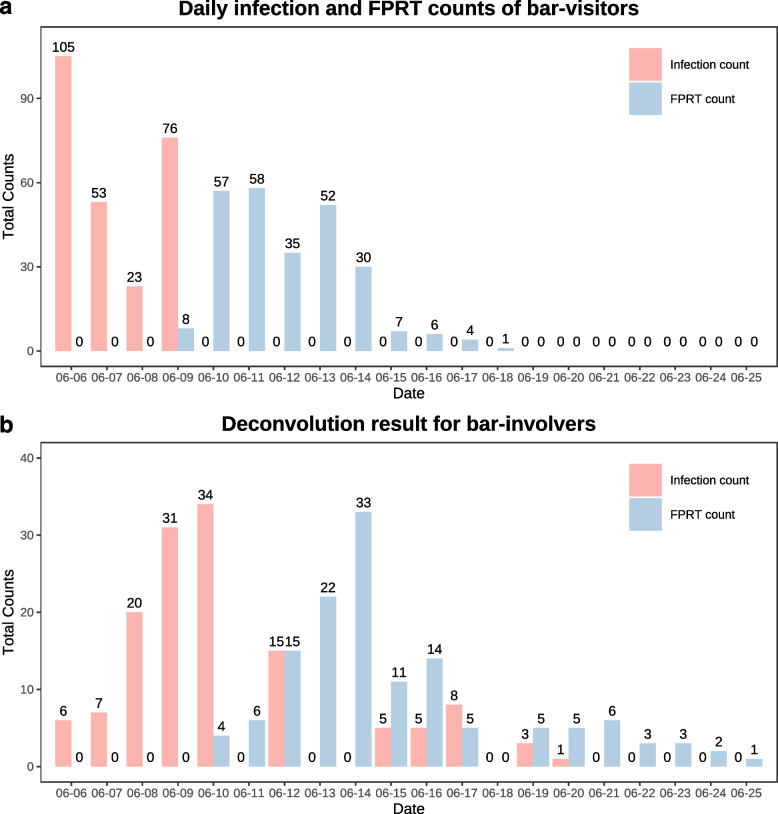
Daily infections and FPRT series of bar-visitors (**a**) and bar-involvers (**b**) compared in the bar epidemic. The daily infection counts are estimated by an E-M algorithm for bar-visitors and deconvolved by the Richard-Lucy algorithm for bar-involvers

### Stability of delay distribution estimate

The reported daily FPRT series were subject to variations caused by both epidemic and technical factors. To study the stability or variability of the estimated delay distribution, we perturbed the observed series and generated re-estimates by the same E-M algorithm. The FPRT series were perturbed in two ways. First, they were self-perturbed, and this technique is known as bootstrap. Namely, 100 simulated FPRT series were re-sampled from the multinomial distribution with the empirical frequencies $(G_1/N,G_2/N,\dots ,G_n/N)$. Second, 100 FPRT series were simulated by introducing a normally distributed noise to the observed series. For each of the simulated series, we implemented Algorithm S1 to obtain an estimation of the delay distribution $\textbf{w}$. The results of the perturbed FPRT series are shown in Fig. 1c. In general, the perturbed delay distribution remained to be unimodal, and the peak was around day 4. Meanwhile, the probabilities of day 4 and day 7 are less stable. We also obtained confidence intervals for the estimated delay distribution from the bootstrapping results. A 95% confidence interval for the mean of delay days is [4.426, 5.545].

### Reconstruction of the daily incidences of the bar-involvers

We further deconvolved the daily infection series of those involvers using the Richard-Lucy algorithm. Specifically, we initialize $\hat{\textbf{F}}$ by setting $\hat{F}_1=\hat{F}_2=\dots =\hat{F}_{n-3}=\dfrac{N}{n-3}$, $\hat{F}_{n-2}=\hat{F}_{n-1}=\hat{F}_n=0$. The deconvolution result is shown in Fig. 2b. The result suggests that the epidemic chain was immediately cut off on June 10, but a leak occurred on June 12. In fact, the Beijing government reacted rapidly upon the report of the first PCR-positive case on June 9. The bar was closed and immediate epidemiological investigation and tracking were implemented within two days. All known visitors were brought to quarantine hotels or hospitals. However, in the local news, three bar-visitors were reported for not following the prevention and control rules [12]. Their activities before the diagnosis of COVID-19 led to the leak we observed. Another leak occurred before June 16 and was not noticed until an infector was detected by the mass RT-PCR test on June 19 [13].

In contrast to non-visitors, the average daily incidence counts of the 258 bar-visitors were imputed (Fig. 2a and Supplementary Notes S1, S2) by the E-M algorithm along the way of parameter estimation. The imputed values of $\textbf{F}_{vis}$ from June 6-9 were their mathematical expectations evaluated at the maximum likelihood estimates of parameters.

The pattern in the daily incidences of the bar-involvers is very different from that of the bar-visitors (Fig. 2a). The bar-involvers were primarily infected by the bar-visitors. Although the incidences of bar-visitors on June 6 and 7 were imputed to be 105 and 53 respectively, the incidences of bar-involvers, who were in direct contact with visitors, were imputed to be merely 6 and 7 on June 6 and 7 respectively. Starting from June 8, they increased sharply. This implies that the transmission risk was relatively low in the first two days after being infected, and increased substantially from day 3 to day 5. Moreover, during the period of June 6 to 26, only one confirmed case was not linked to any contact with bar visitors. This implies that the risk of the secondary contact being infected was much less than that of the direct contact being infected in the outbreak.

### Application of real-time deconvolution to Wuxi City’s epidemic

**Fig. 3:**
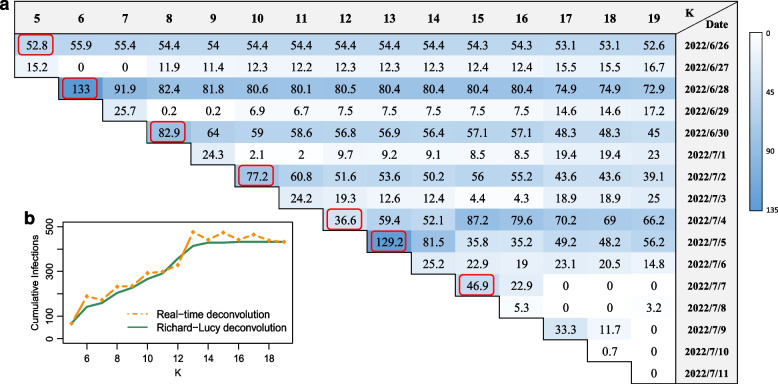
The real-time deconvolution of Wuxi’s epidemic data. **a** The table presents the deconvolved infection counts in Wuxi from June 26 to July 11 obtained by Algorithm 2. The real-time deconvolution using the FPRT counts up to the *K*-th day is shown in the *K*-th column, which includes reconstructed infection counts up to the $(K-3)$-th day. The days with explosive incidence increases are marked by red rectangles. **b** A comparison of the cumulative infection counts up to the $(K-3)$-th day using real-time deconvolution versus Richard-Lucy deconvolution

Right after the outbreak of the Beijing Heaven bar, another one emerged in Wuxi City around June 26th, 2022, caused by imported cases from other provinces after a period of “dynamic-zero”. We collected the daily FPRT cases reported by the Wuxi Government [14] starting from June 26 to July 15 when this outbreak ended. Then we applied the real-time deconvolution algorithm to the FPRT series from June 30 to July 14 ($K=5,6,\dots ,19$, *c.f.* Fig. 3a). Since the two outbreaks were close in timing and in the test pipeline, the delay $\hat{\textbf{w}}$ estimated in Section “The PCR delay distribution of the Omicron variant” was reused in the deconvolution.

Unlike the Richard-Lucy deconvolution, which takes input of the whole data only once, the real-time deconvolution is an online algorithm. After obtaining the *K*-th day FPRT count, it updated estimates of all the incidence counts up to the $(K-3)$-th day. The results of real-time deconvolution are shown in Fig. 3a. The line chart in Fig. 3b shows that the cumulative incidence count resulted from the real-time deconvolution is comparable to that from the Richard-Lucy algorithm. Furthermore, the real-time deconvolution detected all the days marked by explosive incidence increases, namely, the first, third, fifth, seventh, ninth, and tenth day. During the acute phase of the pandemic, the real-time deconvolution can serve as a timely alarm. Since an explosive increase often corresponds to a very recent substantial infection event in the public space, the real-time deconvolution results provide a scientific basis for local disease control authorities to take proper measures such as rapid epidemiological investigations and isolations.

It is worth mentioning that the Wuxi Government’s epidemic prevention and control measures were implemented with good precision, guaranteeing the rapid containment of the outbreak and minimizing its impact on the economy and society.

## Discussion

The delay between the incidence and a positive test result is also observed in infectious diseases diagnosed through other testing protocols such as large-scale antigen testing. If the delay model (1) is approximately valid, we can apply the deconvolution method to reconstructing the incidences. The pipeline of delay function estimation and incidence reconstruction provides a paradigm for studying other epidemic outbreaks based on delayed observed data.

By imposing non-negative constraints, the deconvolution of infection incidences is formulated as a LININPOS model, whose theoretical properties were extensively investigated by Vardi, Lee, Li (the corresponding author of this article) and Speed. The Kuhn-Tucker condition required by the non-negativity works particularly well when the incidences are sparse, that is, the infection events are separate from each other. To obtain sparse results, machine learning techniques usually impose a penalty on the size of the infection incidences [6]. In the case of the Heaven-bar outbreak, we found no further improvement by adding a penalty term. As shown in Fig. 2b, the separation of the infection events could help staff at centres for disease control and prevention conduct epidemiological investigations more efficiently. When the infection events were not sparse, the gain from non-negative constraints would be less significant.

To reconstruct daily infection incidences fairly, we need to estimate the delay function adaptively as the virus evolves. The delay function in the convolution model represents the overall effect of the disease latency, PCR sensitivity, and the operation pipeline performance. It is helpful to know if we can estimate the overall delay function using data from one outbreak with such a sample size of 258 as in the Heaven-bar case. In the Heaven-bar outbreak, other than daily FPRT counts, the infection dates of the bar-visitors were narrowed down to within 3 days and a half. Besides, the outbreak was not confounded with any others. We showed, with the availability of such clean data, the delay function of the Omicron variant can fairly be estimated by maximizing likelihood. The bootstrap method was used to evaluate the variability of the delay function estimate.

The goodness of deconvolution relies on the quality of the delay function. In this study, the delay function estimated from the bar-visitors was applied to the bar-involvers in the same outbreak, and to the Wuxi outbreak occurred right after the Heaven-bar one. The applicability of the delay function to variants other than Omicron and to other countries would be less reliable. Similar data as that of bar-visitors would be helpful in re-estimating the delay function.

The deconvolution result of the Heaven-bar outbreak suggests that over 86% and 93.9% of diagnosed patients can be detected by RT-PCR test within respectively 5 days and 7 days. The third day after infection is a critical time point, from which the infectivity of the infected patients increases sharply. Our deconvolved infection incidences of the bar-involvers suggest that if all close contacts are isolated within 3 or 4 days, the spread chain will be cut off quickly. On June 28, 2022, the Joint Prevention and Control Mechanism of the State Council released the ninth edition of the COVID-19 prevention and control plan, in which the quarantine period for close contacts and inbound travellers has been shortened to “7 + 3”. Our deconvolution results supported the policy adjustment.

In the meantime of estimating the delay function, the algorithm S1 imputed the infection counts for the bar’s epidemic from June 6 to 9 as well. While only 53 and 23 were infected on June 7 and 8, 105 and 76 were infected on June 6 and 9. Notably, exactly on these two days the suspicious “number-zero patient” visited the bar [15]. As reported, this individual had only mild symptoms of COVID-19. This suggested that some infected may be more infectious than others. Epidemic prevention and control needs to pay specific attention to these “super spreaders”.

Right after we finished the mathematical modeling work of deconvolution based on the Heaven-bar epidemic data, another Omicron outbreak occurred in Wuxi. Through a colleague, the findings from the Heaven-bar epidemic data analysis and our prediction based on the deconvolution were passed on to a Wuxi local government official. After 431 PCR positives were reported, the outbreak was successfully controlled within a month. It is expected that participatory mathematical modeling work could be made known to the community and be utilized by experts in other regions and countries timely through international consortia [16, 17].

## Conclusion

The study has two main objectives. The first objective is to reconstruct the series of daily infection count $\textbf{F}$ from the observed counts of $\textbf{G}$, given the delay function $\textbf{w}$. The second objective is to estimate the delay function $\textbf{w}$, which is specific to a variant and the test operation pipeline in a region, from an epidemic outbreak with relatively sufficient contact tracing records and information. To achieve the primary objective, we developed a general deconvolution approach, which is described in detail in Algorithm 1 and Algorithm 2. In addition, we presented an E-M algorithm to estimate the delay function $\textbf{w}$ using the Heaven Supermarket Bar epidemic data.

The proposed deconvolution method is generally applicable to other infectious diseases if the delay model can be assumed to be approximately valid. To ensure a fair reconstruction of daily infection incidences, the delay function should be estimated in a similar context in terms of virus variant and test protocol. Both the delay estimate from the E-M algorithm and the incidences resulted from deconvolution are valuable for epidemic prevention and control. The real-time deconvolution is particularly useful information during the epidemic’s acute phase because it can help the local disease control authorities modify the control measures more promptly and precisely.

### Supplementary Information

**Additional file 1.**

## Data Availability

The datasets supporting the conclusions of this article are included within the article and its additional files.
