# Supplementary material for: Reconstructing COVID-19 incidences from positive RT-PCR tests by deconvolution
Source: BMC Infect Dis. 2023 Oct 11;23:679. doi: 10.1186/s12879-023-08667-1 (PMC10568936; doi:10.1186/s12879-023-08667-1)
Supplement: Supplementary file 1 — Additional file 1. [file 12879_2023_8667_MOESM1_ESM.zip › 12879_2023_8667_MOESM1_ESM/SupplementaryMaterial.pdf]

Supplementary Notes: Reconstructing COVID-19 incidences from positive RT-PCR tests by deconvolution

Mengtian Li, Jiachen Li, Ke Wang, Lei M Li

Infection Date \ FPRT Date	1	2	...	m+1	m+2	...	n-m	n-m+1	...	n	col_sum
1	$N_{1,1}$	$N_{1,2}$	...	$N_{1,m+1}$	0	...	0	0	...	0	F_1
2	0	$N_{2,2}$	...	$N_{2,m+1}$	$N_{2,m+2}$	0	0	0	...	0	F_2
3	0	0	$N_{3,3}$	...	$N_{3,m+2}$	$\ddots$	0	0	...	0	F_3
$\vdots$	$\vdots$	$\vdots$	$\vdots$	$\vdots$	$\vdots$	$\ddots$	$\ddots$	$\ddots$	$\ddots$	$\vdots$	$\vdots$
n-m	0	0	...	...	...	0	$N_{n-m,n-m}$	$N_{n-m,n-m+1}$	...	$N_{n-m,n}$	F_{n-m}
row_sum	G_1	G_2	...	G_{m+1}	G_{m+2}	...	G_{n-m}	G_{n-m+1}	...	G_n	N

Figure S1. The matrix of the count of patients infected on day i and FPRTed on day j, $N_{i,j}$. The matrix is non-negative and sparse. Considering that patients infected on day i are spread to the subsequent m days to be FPRTed, each row has only m non-zero elements(filled with gray). The column sum and the row sum correspond to the daily infection count F_i and the daily FPRT count G_j, respectively.

Supplementary Note S1: MLE of the delay distribution when the complete data is known

Considering the epidemic model based on the patients' infected date $\mathbf{X}(k)$ and reported date $\mathbf{Y}(k)$, if we know the complete paired data: $\{(\mathbf{X}(k), \mathbf{Y}(k)), k = 1, \ldots, N\}$, or the count of patients who were infected on day i and FPRTed on day j, $\{N_{i,j} \triangleq \sum_k I_{(\mathbf{X}(k)=i, \mathbf{Y}(k)=j)}\}$, a traditional MLE can be obtained as follows.

We consider the joint distribution of $\mathbf{Z}_k \triangleq \mathbf{Y}(k) - \mathbf{X}(k), k = 1, \dots, N,$

$$L(\mathbf{w}; (\mathbf{Z}_k)) = \Pi_{k=1}^{N} Pr(\mathbf{Z}_k = t_k) = \Pi_{t=0}^{m} w_t^{\sum_{i=1}^{n} N_{i,i+t}}. \tag{1}$$

Take the logarithm of (1), we have:

$$\begin{aligned} l(\mathbf{w}|(N_{i,j})) &= \sum_{i=1}^{n-m} N_{i,i} \log(w_0) + \sum_{i=1}^{n-m} N_{i,i+1} \log(w_1) + \cdots + \sum_{i=1}^{n-m} N_{i,i+m} \log(w_m) \\ &= \sum_{t=0}^{m} \sum_{i=1}^{n-m} N_{i,i+t} \log(w_t) \end{aligned} \tag{2}$$

Note the boundary condition

$$\sum_{t=0}^{m} w_t = 1, \tag{3}$$

replace w_m by $(1 - \sum_{t=0}^{m-1} w_t)$, and take the derivation of $l(\mathbf{w}, \mathbf{N})$,

$$\frac{\partial l}{\partial w_t} = \frac{\sum_{i=1}^{n-m} N_{i,i+t}}{w_t} - \frac{\sum_{i=1}^{n-m} N_{i,i+m}}{1 - \sum_{t=0}^{m-1} w_t}, \quad t = 0, \dots, m-1. \tag{4}$$

Let $\frac{\partial l}{\partial w_t} = 0$, we obtain the MLE of w_t

$$\hat{w}_t = \frac{\sum_{i=1}^{n-m} N_{i,i+t}}{N}, \quad t = 0, \dots, m. \tag{5}$$

Supplementary Note S2: the E-M algorithm to estimate the delay distribution based on missing data However, the paired information for individuals was missing here. So we adopted the expectation–maximization (E-M) algorithm, which is often used to obtain MLE for models involving latent variables in addition to unknown parameters and known data observations. We first illustrate two conditional distributions that will be used in the E-M algorithm.

Given that the patient is infected on day i, the conditional random variable of his FPRT date, $\mathbf{Y}|\mathbf{X} = i$, obeys a discrete distribution, namely,

$$P(\mathbf{Y} = j|\mathbf{X} = i) = P(\mathbf{Y}(k) = j|\mathbf{X}(k) = i) = w_{j-i}, \quad j = i, i+1, \dots, i+m. \tag{6}$$

Symmetrically, given that the patient is FPRT on day j, the conditional random variable of his infected date $\mathbf{X}|\mathbf{Y}=j$ also obeys a discrete distribution.

$$P(\mathbf{X}=i|\mathbf{Y}=j)=P(\mathbf{X}(k)=i|\mathbf{Y}(k)=j)\triangleq p_{j,i},\quad i=j-m,j-m+1,\dots,j. \tag{7}$$

If we write the probability as the expectation of an indicator variable, then the above equations becomes:

$$E[I_{(X=i,Y=j)}]=w_{j-i}E[I_{(X=i)}],\quad j=i,i+1,\dots,i+m. \tag{8}$$

$$E[I_{(X=i,Y=j)}]=p_{j,i}E[I_{(Y=j)}],\quad i=j-m,j-m+1,\dots,j. \tag{9}$$

Sum over $k\in\{1,\dots,N\}$,

$$\text{for fixed } i:\quad E[N_{i,j}]=w_{j-i}E[F_i],\quad j=i,i+1,\dots,i+m. \tag{10}$$

$$\text{for fixed } j:\quad E[N_{i,j}]=p_{j,i}E[G_j],\quad i=j-m,j-m+1,\dots,j. \tag{11}$$

Therefore, in the E-Step of the E-M algorithm, we impute the conditional probability $p_{j,i}$ by taking the fraction of $E[N_{i,j}]$ over $\sum_i E[N_{i,j}]$, and then impute the daily count $N_{i,j}$ by taking the expectation of the multinomial distribution. In the M-Step, we obtain the MLE of the delay function w and the estimation of daily infection count $\hat{F}_i$. The algorithm is as follows:

Algorithm S1: The E-M algorithm of estimating the delay distribution based on missing data.

1. Initialize $\hat{\mathbf{w}}=(\hat{w}_0,\dots,\hat{w}_m)$, $\hat{\mathbf{F}}=(\hat{F}_1,\dots,\hat{F}_n)$;
2. E-Step:
   (a) impute the conditional probability $p_{j,i}, j=1,2,\dots,n$ by

$$\hat{p}_{j,i}=\begin{cases} 0, & \text{if } i<j-m \text{ or } i>j;\\ \frac{\hat{F}(i)\hat{w}(j-i)}{\sum_{i=j-m}^{j}\hat{F}(i)\hat{w}(j-i)}, & \text{if } j-m\le i\le j. \end{cases} \tag{12}$$

(b) impute the complete data $\hat{N}_{i,j} = G_j \hat{p}_{j,i}$;

3. M-Step:

(a) compute the MLE according (5): $\hat{w}_{t,new} = \frac{\sum_{i=1}^{n} \hat{N}_{i,i+t}}{N}, \quad t = 0, \ldots, m.$

(b) for $i = 1, \ldots, n-m$, update $\hat{F}_i$ by

$$\hat{F}_{i,\text{new}} = \sum_{j=1}^{n} \hat{N}_{i,j}. \tag{13}$$

Since E-M algorithms are sensitive to initialization, we initialize $\hat{\mathbf{w}}$ and $\hat{\mathbf{F}}$ by optimizing the information of the dataset. On the one hand, we initialize $\hat{\mathbf{F}}$ by $(\frac{2}{7}, \frac{2}{7}, \frac{2}{7}, \frac{1}{7})$ since the Heaven Supermarket bar opened for only 4 days and shut down on the noon on the fourth day. On the other hand, we initialize $\hat{\mathbf{w}}$ by $\hat{w}_0 = \hat{w}_1 = \hat{w}_2 = 0, \hat{w}_3 = \cdots = \hat{w}_9 = \frac{1}{7}$ for the message that the first patient was not detected until June 9, three days after the epidemic outbreak, while a strict 48-hour nucleic acid inspection system on the public was running at that time.

Supplementary Note S3: The Kuhn-Tucker condition of the Richard-Lucy deconvolution problem The deconvolution problem can be solved as an optimization problem subject to nonnegativity constraints. We consider the following problem:

$$\begin{aligned} &\underset{\mathbf{f}}{min}\, D_{KL}(\mathbf{g}||\mathbf{Wf}) \\ &subject\ to\!:\ f_i \geq 0\ and\ \sum_{i=1}^{n} f_i = 1. \end{aligned} \tag{14}$$

The Kuhn-Tucker condition is:

$$\begin{cases} f_i^* = 0, & \sum_k \frac{w_{i,k} g_k}{\sum_s w_{s,k} f_s^*} < 1,\ for\ i \in \mathcal{E}, \\ f_i^* > 0, & \sum_k \frac{w_{i,k} g_k}{\sum_s w_{s,k} f_s^*} = 1,\ for\ i \in \mathcal{S}. \end{cases} \tag{15}$$

where $\mathcal{E}$ and $\mathcal{S}$ form a partition of the set $\{1, 2, \ldots, n\}$.

Supplementary Note S4: The Kuhn-Tucker condition of the real-time deconvolution problem The real-time deconvolution problem can also be formalized as a convex optimization problem as follows:

$$
\begin{aligned}
&\underset{\mathbf{f}_{1:K}}{min}\, D_{KL}(\mathbf{g}_{1:K}||\mathbf{W}\mathbf{f}_{\mathbf{1:K}}) \\
&\textit{subject to: } f_i \geq 0 \textit{ and } \sum_{i=1}^{K}(\sum_{t=0}^{K-i} w_t) f_i = 1.
\end{aligned}
\tag{16}
$$

The corresponding Kuhn-Tucker condition can be derived as follows:

$$
\begin{cases}
f_i^* & = 0, \quad \sum_k \frac{w_{k-i} g_k}{(\sum_{t=0}^{K-i} w_t) \sum_s w_{k-s} f_s^*} < 1, \ \textit{for } i \in \mathcal{E}, \\
f_i^* & > 0, \quad \sum_k \frac{w_{k-i} g_k}{(\sum_{t=0}^{K-i} w_t) \sum_s w_{k-s} f_s^*} = 1, \ \textit{for } i \in \mathcal{S}.
\end{cases}
\tag{17}
$$

where $\mathcal{E}$ and $\mathcal{S}$ form a partition of the set $\{1, 2, \ldots, n\}$.
